# Targeting Virulence Genes Expression in *Vibrio vulnificus* by Alternative Carbon Sources

**DOI:** 10.3390/ijms232315278

**Published:** 2022-12-03

**Authors:** Aldo Nicosia, Monica Salamone, Marcello Tagliavia

**Affiliations:** 1 Institute for Biomedical Research and Innovation, Italian National Research Council (IRIB-CNR), 90146 Palermo, Italy; 2 Department of Biological, Chemical and Pharmaceutical Sciences and Technologies (STEBICEF), University of Palermo, 90128 Palermo, Italy

**Keywords:** *Vibrio vulnificus*, virulence genes expression, virulence inhibitors, carbon sources, dual function small RNA, VvdRP

## Abstract

*Vibrio vulnificus* is an opportunistic human pathogen causing self-limiting gastroenteritis, life-threatening necrotizing soft tissue infection, and fulminating septicaemia. An increasing rate of infections has been reported worldwide, characterized by sudden onset of sepsis and/or rapid progression to irreversible tissue damage or death. Timely intervention is essential to control the infection, and it is based on antibiotic therapy, which does not always result in the effective and rapid blocking of virulence. Inhibitors of essential virulence regulators have been reported in the last years, but none of them has been further developed, so far. We aimed to investigate whether exposure to some carbon compounds, mostly easily metabolizable, could result in transcriptional down-regulation of virulence genes. We screened various carbon sources already available for human use (thus potentially easy to be repurposed), finding some of them (including mannitol and glycerol) highly effective in down-regulating, in vitro and ex-vivo, the mRNA levels of several relevant -even essential- virulence factors (*hlyU*, *lrp*, *rtxA*, *vvpE*, *vvhA*, *plpA*, among others). This paves the way for further investigations aiming at their development as virulence inhibitors and to unveil mechanisms explaining such observed effects. Moreover, data suggesting the existence of additional regulatory networks of some virulence genes are reported.

## 1. Introduction

*Vibrio vulnificus* is a halophilic Gram-negative bacterium that inhabits preferentially warm estuarine and marine environments worldwide and is much less abundant in seawater with higher salinity. However, due to global warming, there are concerns about the increasing number of cases of infection because of its faster growth in warm seawater [1,2,3,4]. *V. vulnificus* is considered an opportunistic human pathogen [5], that may cause three types of infections: (1) acute gastroenteritis, (2) primary and secondary sepsis, and (3) necrotizing wound/soft tissues infections. Gastroenteritis, associated with the ingestion of contaminated, uncooked seafood, remains frequently unreported, because it is usually overlooked/undiagnosed, self-limiting, and rarely associated with symptoms requiring medical care. The lethality following ingestion is due to septicaemia (often without gastroenteritis), following bacterial dissemination through the bloodstream; it accounts for over 95% of recorded deaths due to foodborne infections, making *V. vulnificus* the most lethal food-borne pathogen [6]. Primary sepsis is the most life-threatening clinical event in *V. vulnificus* infection, with a mean mortality rate exceeding 50%, which makes this bacterium as lethal as category BSL 3 and 4 pathogens [5]. Wound infections are less frequent and often caused by direct contact with open wounds or scraped skin with contaminated seawater or materials [7]. The infection can rapidly evolve into severe necrotic lesions, which may further progress to secondary septicaemia. Extensive tissue lesions (necrotizing fasciitis) require surgery (fasciotomy, debridement, and even limb amputation), often essential to eliminate the pathogen and limit the spread of bacterial toxic molecules (responsible for most long-lasting tissue-damaging activities).

*V. vulnificus* rarely infects healthy individuals, and most clinical cases (especially primary sepsis) are from patients with underlying diseases, including hepatic disorders, diabetes, and iron overload, as well as immunocompromised ones. Moreover, the infection is much more frequent in males (aged > 40) than in females [5]. However, over 80% of *V. vulnificus* wound infections occur in healthy people, which suggests that, in the two routes of infection, different pathogenic mechanisms are involved [8].

Several virulence genes preferentially expressed during infection have been identified, among which global virulence regulators, including *lrp*, *smcR*, *hlyU* and *aphB* [9,10,11,12,13] involved in the expression of several downstream virulence factors genes, such as *plpA* (phospholipase A_2_) [14], *rtxA* (multifunctional-autoprocessing repeats-in-toxin (MARTX) toxin) [15,16], *vvhA* (cytolysin) [17] *vvpE* (elastase), as well as genes that regulate iron uptake, like *vvuA* (involved in vulnibactin biosynthesis) and *hupA* (heme receptor protein) [9].

The very short incubation time (even less than 16 h) in wound infection, often followed by rapid dissemination and even fulminating septicaemia, as well as the sudden onset of symptoms warrant timely medical intervention, based first on antibiotic therapy.

However, the administration of antimicrobials in septic patients may result in even fatal side effects, due to the release of toxic molecules from dead bacteria into the bloodstream [18]. Moreover, the increasing frequency of antibiotic-resistant isolates worldwide may make empirical antibiotic therapy challenging. Such issues have prompted the search for alternative approaches, based on non-antimicrobial inhibitors of virulence factors such as HlyU, rather than antimicrobial chemotherapy [19,20]. Such a strategy would overcome antimicrobial resistance and has a very low risk of selection of resistant strains, without compromising pathogen isolation. Similarly to antibiotics, such compounds are expected to exert maximum effects if administered in the early phases of infection. However, although much attractive, these drugs have been tested in vitro, but none of them -to our knowledge- has undergone further development. In this scenario, the possibility of attenuating the virulence using compounds already available and approved for human/clinical use and with excellent safety profiles, would facilitate their translation to the clinics. Moreover, this might pave the way to the setup of innovative therapeutic protocols that might revolutionize the emergency treatment when *V. vulnificus* infection is ascertained or only suspected. The close link between environmental conditions and virulence is well-established, and it is reasonable to hypothesize that the involvement of the cAMP Receptor Protein (CRP) in the transcriptional regulation of several virulence factors might be a relevant bridge between carbon source availability and virulence [21]. Moreover, a small dual-function RNA, named VcdRP and regulated by CRP, has been recently identified in *V. cholerae* as involved in toxin production, carbon uptake and regulation of global metabolism [22]. A nearly identical *locus*, mapping on chromosome 1 of *V. vulnificus*, has been reported [22], though it has not been investigated, so far. Based on such knowledge, as well as on previous data about human pathogenic bacteria closely related to *V. vulnificus* (namely, *V. parahaemolitycus* and *V. alginolyticus*) [23], we aimed to investigate whether selected chemicals (suitable for, or already in clinical use, also in the setting of sepsis) could down-regulate the most relevant virulence genes. Using a clinical isolate *V. vulnificus* type strain, we investigated the effects of various carbon sources on the expression levels of a selected panel of representative virulence genes, including essential ones, both in vitro and ex vivo. Compounds able to strongly affect the expression of relevant virulence genes were identified, which paves the way for future pre-clinical and clinical development.

## 2. Results

We have previously reported that some carbon sources were able to alter the pattern of secreted proteases in *V. parahaemolitycus* and *V. alginolyticus* [23]. Based on the knowledge that the expression of virulence factors of *V. vulnificus*, including secreted proteins, is highly dependent on environmental stimuli, and that the host-pathogen interaction implies some metabolic rewiring in bacteria, we wondered if some carbon sources (selected among those compatible with possible clinical uses) could affect the expression of key virulence genes.

To obtain indications about any detectable influence of carbon sources on the bacterium physiology, we exploited the proteolytic secretome as a possible proxy of metabolic effects. Such an approach allowed for a preliminary selection of conditions that could justify further investigations at the gene-expression level.

**Figure 1:**
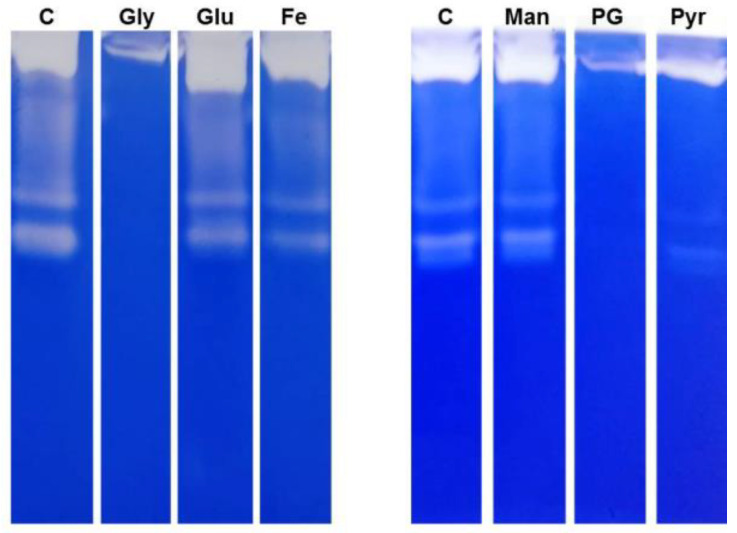
Secreted proteases from *V. vulnificus* grown with different carbon sources. The gelatinolytic activity of supernatants from liquid cultures of *V. vulnificus* was analyzed by zymography using gelatine as substrate in presence of CaCl_2_. Culture media were as follows: MB (C), MB supplemented with glycerol (Gly), glucose (Glu), FeCl_2_ (Fe), mannitol (Man), propylene glycol (PG), or sodium pyruvate (Pyr). Loading volumes were normalized with measured cell density (OD_600_).

Similarly to what was obtained in *V. parahaemolitycus* and *V. alginolyticus*, the growth in presence of glycerol resulted in a strongly altered pattern of secreted proteases, most of which disappeared (Figure 1). Based on such a result, we aimed to explore the effects of other carbon sources, all having in common ease of uptake and utilization, namely propylene glycol (chemically related to glycerol), mannitol (a clinically employed polyol), pyruvate (the glycolysis end-product, clinically employed as ethyl-ester), and maltose, most of which showed to affect the proteolytic-enzymes pattern (Figure 1).

This prompted us to assess if these carbon sources (including trehalose, which has been reported [24] to affect the virulence of some bacteria) could affect the expression of a set of selected virulence genes, including global virulence regulators (namely *lrp, smcR, hlyU, aphB*) [9,10,11,12] essential effectors such as *plpA* and *rtxA* [14], as well as other relevant factors including *vvhA, vvpE, vvuA, vvA1308* (siderophore ABC transporter)*,* and *hupA* [15,17].

To verify the suitability of the experimental culture conditions to reliably assess the responses, we firstly tested the effect of two conditions, namely glucose or iron supplementation, well-known to be involved in virulence and affect gene expression [16,25].

**Figure 2:**
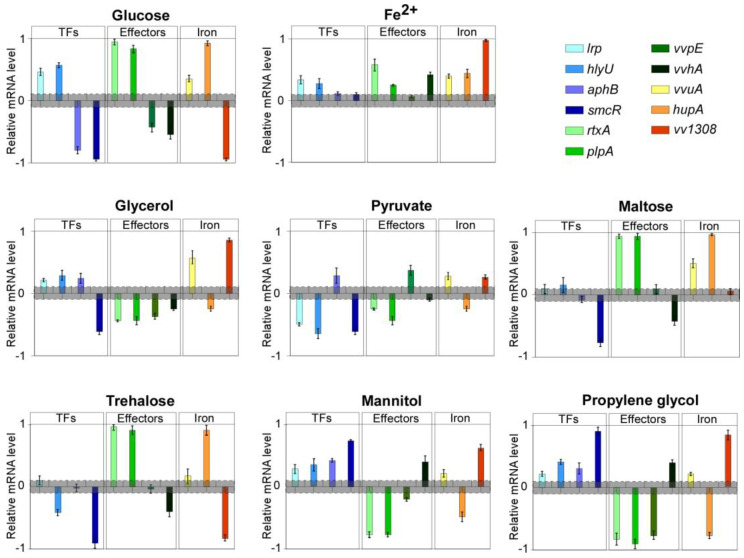
Carbon sources differentially affect the expression of virulence genes in *V. vulnificus*. RT-qPCR results showing the mRNA levels of indicated genes in *V. vulnificus* after growing in presence of a selected carbon source with respect to *gyrB* and *recA*. Data are reported as a 10log scale, bars represent mean ± SD, values beyond the gray zone were considered statistically significant at *p* ≤ 0.05. Statistical analyses by Student’s *t*-tests.

Relevant variations were detected in the expression level of several genes in both cases. In detail, the up-regulation of *hlyU*, *rtxA*, *plpA*, *lrp*, *hupA* and *vvuA* (between 2 and 9-fold), and the down-regulation of *vvhA*, *vvpE*, *vva1308*, *aphB* and *smcR* (from 5 and 8-fold lower than the control) was observed in cells grown with glucose (Figure 2).

In contrast, iron supplementation did not result in gene down-regulation, compared with the control, whereas *hlyU*, *vvhA*, *rtxA*, *plpA*, *lrp*, *hupA*, *vva1308* and *vvuA* were, to a different extent (between 2 and 10-fold), upregulated, consistently with what reported in iron overload settings [21] and with the siderophilic behavior of the pathogen (Figure 2). The latter results suggested that, despite the culture medium being far from resembling the host-pathogen interaction, the bacterium retains its ability to orchestrate a consistent response to environmental stimuli and to modulate its virulence gene expression profile accordingly. This led us to consider the experimental system fully suitable for our aims.

### 2.1. Carbon Sources Differentially Affect Virulence Genes Expression

In the subsequent experimental step, that panel of genes was evaluated for variations in the expression levels in bacteria grown with supplementation of either glycerol, propylene-glycol (chemically closely related to glycerol), mannitol, sodium pyruvate, maltose, or trehalose (Figure 2). These compounds are all available for use in humans, either for clinical application or as food additives.

Glycerol supplementation resulted in a marked down-regulation of virulence genes like *vvhA, rtxA, plpA, vvpE, hupA* and *smcR.* Conversely, essential regulators like *hlyU, lrp,* and *aphB* were up-regulated (fold change of 2.9, 1.8, and 2, respectively), as well as *vvuA* and *vva1308* (6.1 and 9.2 folds, respectively).

The growth with sodium pyruvate resulted in significant down-regulation of *hlyU, plpA, rtxA, lrp, hupA,* and *smcR* (from 3 to 5-fold lower than the control), while *vvpE, vvuA, aphB*, and *vva1308* mRNA levels were increased of approximately two folds.

Maltose supplementation induced down-regulation of *vvhA* and *smcR* (at least 4-fold lower), while resulting in a marked up-regulation of *rtxA, plpA, hupA* (about 9-fold), and *vvuA* (3-fold). The mRNA levels of all the other genes were substantially unchanged.

Growth with trehalose resulted in the strong down-regulation of *vva1308* and *smcR* (about 8-fold lower), as well as of hlyU and *vvhA* (about 3-fold lower), and in the marked up-regulation of *rtxA, plpA, and hupA* (from 7 to 10-fold), while the level of the other genes was nearly unchanged.

The variation in the transcriptional profiles observed with the supplementation of either propylene-glycol and mannitol were overall overlapping and showed a very marked down-regulation of *hupA* and, notably, of the three main secreted virulence factors, namely *rtxA, plpA, and vvpE* (about 8-fold lower than the respective controls), whose levels dropped down near to the detection limit. Conversely, *vvuA, lrp, hlyU, vvhA, aphB, vva1308*, and *smcR* were upregulated (from 2 to 9-fold).

### 2.2. Combining Carbon Sources to Target Virulence Genes

**Figure 3:**
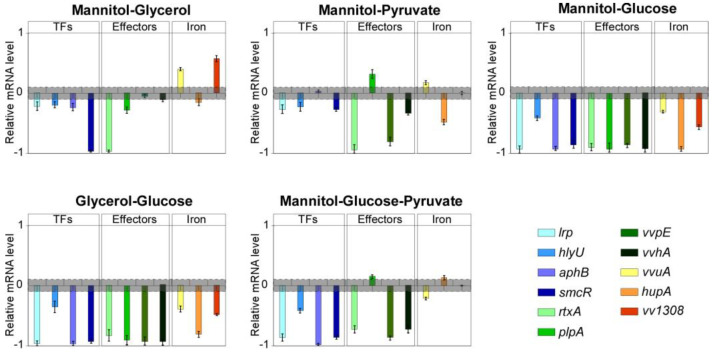
Combination of different carbon sources target the virulence genes expression in *V. vulnificus*. RT-qPCR results showing the mRNA levels of indicated genes in *V. vulnificus* after growing in presence of mannitol, glycerol, pyruvate, and glucose combinations with respect to *gyrB* and *recA*. Data are reported as 10log scale, bars represent mean ± SD, values beyond the gray zone were considered statistically significant at *p* ≤ 0.05. Statistical analyses by Student’s t-tests.

In the light of the results obtained and having as a goal the maximum down-regulation of the genes encoding the virulence factors actively involved in the bacterial invasion/dissemination and tissue damage (e.g., *rtxA, vvpE, plpA*), and their regulators (among which *lrp* and *hlyU*), we tested combinations of the compounds that had shown the most promising results, namely glycerol, mannitol, and sodium pyruvate (Figure 3).

The combination of mannitol and glycerol resulted in a very strong down-regulation of *smcR* and *rtxA* (at least 10-fold lower than the control); a significant decrease in the expression levels was observed also for *lrp, hlyU, plpA*, and *aphB* (about 2-fold lower). In contrast, *vva1308* and *vvuA* were strongly upregulated (5.8 and 4.5-fold, respectively), while levels of *vvpE*, *vvhA*, and *hupA* were negligibly decreased.

The combination of mannitol and pyruvate resulted in the strong down-regulation of *vvpE* and *rtxA* (8 and 10-fold lower than the control, respectively), and *lrp, hlyU, vvhA*, *hupA* and *smcR* levels decreased significantly (about 2.5-fold lower than control), as well. Conversely, *plpA* was upregulated (2-fold), while *aphB* and *vva1308* were nearly unaffected.

Moreover, as glucose is naturally available in vivo, we tested each compound and some combinations in presence of glucose.

Either glycerol or mannitol, combined with glucose, induced an overall strong down-regulation (about 10-fold lower than the respective controls) of all the analyzed genes, which was less marked for *vvuA, hlyU*, and *vva1308* (between 2 and 5-fold lower than the control). Combination of mannitol with pyruvate in presence of glucose resulted in the down-regulation (between 2 and 10-fold lower than the control) of *smcR, aphB, vvhA, vvpE, lrp, rtxA, vvuA, lrp,* and hlyU, while *plpA* and *hupA* were upregulated (up to 1.6-fold); *vva1308* levels were unaffected.

### 2.3. Carbon Sources Can Target Virulence Genes Expression in Murine Spleen Ex Vivo

This promising scenario would have required some validation by in vivo experiments. However, though several insects (including *Galleria mellonella* larvae) have been proposed as model system to assess the virulence of emerging pathogens [26], they were unsuitable to test the effects of either glycerol or mannitol, because in insects these compounds are toxic [27] or metabolizable [28], respectively.

These limits, along with restrictions in the use of living vertebrates, prompted us to investigate ex-vivo (murine spleen) the effects of some selected compounds. Even though the spleen is not a primary infection site, it is thought to play a critical role in the dissemination of the infection, and the presence of *V. vulnificus* in the spleen after experimental infection has been shown to be associated to fatal outcome in murine models [29].

**Figure 4:**
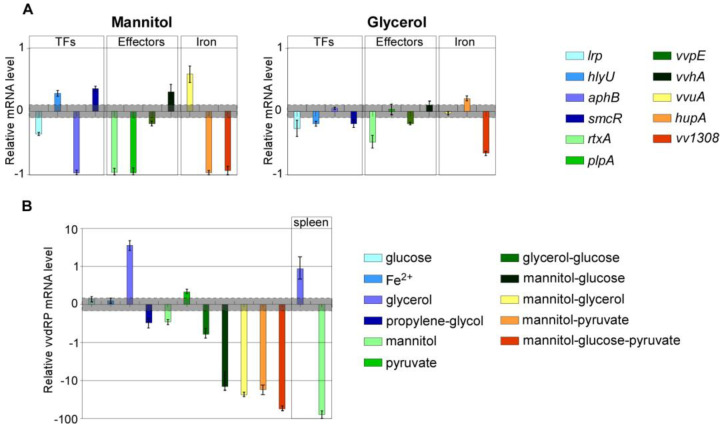
Carbon sources differentially affect the expression of virulence genes in *V. vulnificus* in the murine spleen. (**A**) RT-qPCR results show the mRNA levels of indicated genes in *V. vulnificus* in ex vivo experiments in presence of mannitol or glycerol. (**B**) The expression profile of *VvdRP* under the different conditions analyzed herein. Data are reported as 10log scale, bars represent mean ± SD, values beyond the gray zone were considered statistically significant at *p* ≤ 0.05. Statistical analyses by Student’s *t*-tests.

Based on the results obtained in vitro, we decided to test in the experiments ex vivo only glycerol and mannitol (Figure 4A). Indeed, no combination resulted in inhibitory performances exceeding that of the single compounds. In fact, glycerol and mannitol were the most effective single agents, able to induce overall strong down-regulation in genes encoding both global regulators and toxic enzymes, so that we focused on them to assess their effectiveness also ex vivo.

Following mannitol supplementation in the infected spleen, a significant upregulation of *smcR, vvhA, vvuA* and *hlyU*, with fold changes ranging from 3.7 to 5.8, was observed. Conversely, a very strong down-regulation of *hupA, rtxA, plpA, aphB*, and *vva1308* (about ten-fold lower than control), as well as *lrp* and *vvpE* (2.5 and 1.4 folds lower than control, respectively), was achieved.

Notably, among the most down-regulated genes there are the essential virulence global regulator *lrp*, as well as the three major toxic virulence factors, namely *rtxA*, *plpA*, and *vvpE,* involved in tissues damage and necrosis, and bacterial dissemination.

The treatment with glycerol induced the upregulation of *hupA* (2.3-fold), while most of the major global regulators such as *lrp, hlyU, smcR* were down-regulated, as were *rtxA, vvpE,* and *vva1308* (fold reduction ranging from 2 to 10).

### 2.4. The Dual Function RNA VvdRP Is Expressed in V. vulnificus

In a scenario in which complex interactions might be involved in the differential regulation of various cellular functions, including virulence, we wondered if a possible link between carbon source availability/metabolism and virulence could involve a small dual-function RNA, named *VcdRP*, recently described in *V. cholerae* as involved in the regulation of global carbon uptake and metabolism [22], also through the regulation of PTS, and whose expression is regulated by CRP. The latter factor, in *V. vulnificus,* also regulates the expression of the insulin-degrading enzyme, involved in bacterial growth and dissemination in the bloodstream [30].

An ortholog (herein referred to as *VvdRP*) maps in the genome of *V. vulnificus*, but neither its actual expression nor its possible involvement in the response to carbon sources has been investigated in this pathogen, so far.

Specific primers targeting the 5′ moiety of the *VvdRP* transcript, upstream of the cleavage sites (based on the mapping available for *VcdRP*) involved in RNA processing, were used in RT-qPCR assays to assess the total RNA levels -if any- in all the experimental conditions (Figure 4B). The expression level in cells at the steady state was unchanged in presence of glucose, respect to the control. Instead, with glycerol, substantial increase in the RNA levels (up to 36-fold) was observed. Except for pyruvate, which induced upregulation of 2-folds, in all experimental conditions, a substantial down-regulation of *VvdRP* was observed (Figure 4B).

In ex vivo experiments, glycerol induced *VvdRP* (10-fold upregulation compared to the control), while in presence of mannitol a significant down-regulation was measured (Figure 4B).

No obvious correlation could be found between the trend observed in *VvdRP* levels and that of specific virulence regulators, while down-regulation of *vvpE* (except for the combination of glycerol and mannitol) and *rtxA* seems to correlate with that of *VvdRP*.

## 3. Discussion

We have reported the possibility of targeting the mRNA levels of virulence genes in *V. vulnificus* by providing alternative carbon sources.

The obtained results clearly show that all the tested compounds differentially affected, to various extent, the expression level of many virulence genes, including essential ones (e.g., *plpA*, *hlyU*, *lrp*), and that the exoproteome cannot be considered as a proxy of potential effectiveness of the compounds.

Moreover, variations in the mRNA levels of transcriptional regulators (namely *hlyU*, *lrp*, *smcR, aphB*) may not necessarily result in changes in the levels of downstream genes. No common trend emerged across the various conditions, except for the substantially overlapping effects obtained with glycerol and mannitol, with the latter impairing the expression levels of the most relevant virulence factors involved in the early steps of the infection. As mannitol is reported as not fermentable by the employed strain, this makes us hypothesize some effect not dependent on carbon metabolism.

Pyruvate failed to outperform other compounds in combinations. Notably, both mannitol and pyruvate (also as ethyl-ester) are routinely used in clinical use for the treatment of several—even life-threatening—conditions.

A mechanistic explanation of the pathways involved in the observed effects is difficult to hypothesize, as all such compounds (except mannitol, in the experimental strain) enter glycolysis, which should make the energetic balance itself unlike to be invocated as responsible for the transcriptional effects. Conversely, some signaling associated with the transport/uptake of these compounds (like the phosphoenolpyruvate phosphotransferase system (PTS), well described in *Enterobacteriaceae*) might be hypothesized [31]. However, the strong repressive effect induced by glycerol cannot be explained with the direct involvement of transport systems, as it enters cells by passive diffusion. This makes hypothesize some direct and/or indirect metabolic effects that, in turn, result in specific transcriptional responses. However, indirect effects on PTS and/or downstream signaling cannot be excluded.

Interestingly, the combinations of compounds (e.g., mannitol-glycerol, mannitol-pyruvate) resulted neither in additive nor overlapping effects, but in unique transcriptional profiles, which suggests some interplay between different signaling pathways specifically affected by each carbon source.

Toward the perspective of finding treatments suitable for clinical use, the results obtained in both in vitro and ex vivo experiments indicate that providing alternative carbon sources can effectively alter the mRNA levels of many virulence genes, which in some instances dropped down almost to the detection limit.

Our analyses do not provide information about the exact mechanisms involved in such effects, in which either transcriptional or post-transcriptional regulatory mechanisms might be involved. However, the involvement of mechanisms affecting the mRNA stability is unlikely, as we analyzed transcriptional profiles in cells in the stationary growth phase, after long term exposure to the experimental chemicals. Moreover, the possible interplay between several factors/pathways should be considered.

Though we have shown, for the first time, that the dual function RNA *VvdRP* is expressed in *V. vulnificus* and its levels vary in response to carbon sources, it is unlikely to be involved in the observed effects. Moreover, its expression in *V. vulnificus* showed differences compared with what is reported in *V. cholerae*, in which growth with glycerol does not induce variations in its RNA levels [22]. However, it is worth noting that the data between the two species are not fully comparable, because different culture media were employed, and different quantification methods have been used. Additionally, direct effect on virulence genes appeared unlike based on in silico analyses, as none of the considered transcripts harbor the consensus sequence nearby the translation start codon, which has been reported as essential for the regulation mediated by *VcdRP*. This made us exclude possible direct interactions. Whatever the mechanism(s) responsible for the observed strong reduction in the mRNA levels of the analyzed genes, it is reasonable to hypothesize that these variations might impair the virulence of the bacterium, reducing the production of virulence factors and, in turn, its ability to induce tissue damage and to rapidly disseminate. Although ex vivo experiments cannot provide information about these relevant aspects, the strong molecular responses observed within the relatively short experimental time (3 h) suggest that, also in vivo, the administration of inhibitory compounds could result in a rapid down-regulation of these relevant virulence genes.

Based on the results obtained both in vitro and ex vivo, mannitol proved to be the most promising compound, worth being tested in vivo in preclinical models. The relevant down-regulation exerted on genes encoding essential effectors like PlpA, VvpE, and RtxA (known to be expressed in low-glucose conditions, typical of the dissemination phase after intestinal invasion [32]), despite the upregulation of VvhA, makes hypothesize a relevant impairment of the virulence, reduced production of diffusible toxic molecules (whose effects persist even after bacterial inactivation), possibly resulting in overall improved clinical outcomes.

Mannitol is already largely employed in various clinical settings, which would make easier its “repurposing” as a supportive/antimicrobial drug. This might help to speed up the development of therapeutic approaches aiming firstly at minimizing tissue damage (in worst cases culminating in amputations) and limiting sepsis (the main cause of death), using immediately available drugs. Moreover, this might overcome the gap between the finding of anti-virulence drugs and their clinical development.

Interestingly, our data obtained in various conditions do not fit completely with some regulatory mechanisms proposed for the regulation of virulence factors such as *rtxA* [16].

The expression of *rtxA* has been shown to involve regulatory factors like HlyU, Lrp, H-NS, and CRP, whose interplay seems to explain its regulated expression in response to environmental conditions. Lrp acts as a positive regulator through the binding to specific consensus sequences located in the *rtxA* promoter, while HlyU relieves the repression mediated by H-NS [33]. As the expression of *hlyU* and *lrp* is repressed by CRP, this mechanism represents a link between carbon sources availability, metabolism, and virulence [16].

In contrast to this proposed regulatory network, where the *rtxA* expression should be expected to be linked to the expression levels of its regulators, namely *hlyU* and *lrp*, our data showed some uncoupling between them. In fact, the highest upregulation of *rtxA* was associated with the down-regulation of at least one of its positive regulators, while their upregulation did not result in increased levels of *rtxA* RNA, but even in its downregulation.

Such observations strongly suggest the existence of additional or alternative regulatory mechanisms, which allow for independent *rtxA* transcriptional regulation, worthy of further investigations.

In conclusion, the data herein reported show that some compounds (e.g., mannitol and glycerol) can strongly reduce the mRNA levels of several virulence genes, paving the way for further clinical development in antivirulence therapeutic protocols [32], though the actual effects on virulence have to be addressed, as well as the in vivo effectiveness. Further investigations shall unveil the mechanisms involved in the observed effects.

## 4. Materials and Methods

### 4.1. Strains and Culture Media

*V. vulnificus*, Type strain DSM10143 (from DSMZ, Braunschweig, Germany), was employed in all experiments. Marine Broth (MB, Laboratorios CONDA, Madrid, Spain), Marine Agar (MB containing 1.5% Agar), and TCBS Agar (Laboratorios CONDA, Madrid, Spain) were used for strain maintenance and routine cultivation. MB was used as the standard medium for experiments, and it was supplemented with either FeCl_2_. EDTA (10 µM), glucose (0.4%), maltose (0.5%), trehalose (0.5%), glycerol (0.4%), mannitol (0.5%), propylene-glycol (0.5%), or sodium pyruvate (0.5%), when indicated.

Liquid cultures were inoculated by 1:2000 dilution of an exponentially growing culture in Marine Broth (Laboratorios CONDA, Madrid, Spain) and grown at 37 °C with orbital shaking (about 200 rpm) for 16 h. Then, cultures were chilled in a water-ice bath for 5 min, a small aliquot withdrawn for bacterial count onto selective medium (TCBS) as control, then bacteria were harvested by centrifugation at 5000× *g* for 10 min. Supernatants were analyzed by zymography, and the pellets were immediately frozen and stored at −80 °C or immediately lysed for RNA extraction. Experiments were repeated three times.

### 4.2. Ex Vivo Experiments

Exponentially growing bacterial cells, washed twice in PBS, were put on mouse spleen slices (8 × 10^6^ cfu/200 mg spleen), incubated at 37 °C for 1 h to allow further growth/invasion, then the selected compounds were added to reach the specific concentration, then incubated for further 3 h at 37 °C; then RNA was extracted.

### 4.3. SDS Electrophoresis and Zymography

The analyses were performed following the procedure described in Salamone et al., 2019 [23]. Briefly, aliquots of each bacterial culture supernatants, normalized to OD_600_, were analyzed by gelatin zymography. Separation was performed on 7.5% polyacrylamide gel containing 1 mg/mL bovine gelatin, under non-reducing conditions [34]. After electrophoresis, gels were incubated for 24 h at 37 °C in activation buffer (2 mmol/L CaCl_2_, Tris-HCl 50 mmol/L (pH 7.4), 1.5% Triton X-100, 0.02% Sodium Azide, CaCl_2_ 2 mM). After incubation, gels were stained using Coomassie Brilliant Blue G-250. All chemicals were purchased from Sigma-Aldrich, Milan, Italy.

### 4.4. RNA Extraction and RT-qPCR

RNA was extracted from the pellet obtained by centrifugation of 3 mL of bacterial cultures or from 100 mg of infected and non-infected (as negative control) tissue (for ex-vivo experiments) using TRIzol (ThermoFisher Scientific, Monza, Italy) according to the manufacturer’s instructions. RNA concentration and purity were verified using NanoDrop™ 2000 (ThermoFisher Scientific, Monza, Italy). RNA integrity was evaluated on 1.5% agarose gel. RNA (250 ng) was digested with DNase I (ThermoFisher Scientific, Monza, Italy) to remove DNA contamination, and inactivated by adding 25 mM EDTA. First-strand cDNA was synthesized from 125 ng DNase I-treated RNA using SuperScript cDNA Synthesis Kit (ThermoFisher Scientific, Monza, Italy), according to the manufacturer’s instructions. The cDNAs were tested by PCR using *recA* and *gyrB* primer pairs (Table S1) and diluted 1:10 before use in qPCRs.

The qPCRs were carried out in triplicate, using the BlasTaq2X qPCR MasterMix (Applied Biological Materials Inc., Richmond, BC, Canada), in a 10 µL mixture containing 1 µL of a 1:10 dilution of the cDNAs, in the BIO-RAD CFX96 system, using the following thermal profile: 95 °C for 3 min, 40 cycles of 95 °C for 15 s, and 60 °C for 60 s, and melting curve from 65 to 95 °C. The specific gene amplification was confirmed by agarose gel electrophoresis.

Primer sequences are listed in Table S1, and amplicons length ranged from 100 to 180 bp. The *gyrB* and *recA* were chosen as reference genes, and a normalization factor was calculated as reported in [35,36]. Data analysis was carried out using the ΔΔCT method [37].

Significant differences between values of the different groups and the control group were determined by *t*-test using Statistica 6.0 (StatSoft, Tulsa, OK, USA). The *p* values less than 0.05 were considered statistically significant.

## Data Availability

Not applicable.

## Supplementary Materials

The following supporting information can be downloaded at: https://www.mdpi.com/article/10.3390/ijms232315278/s1.
