# Supplementary material for: Targeting Virulence Genes Expression in Vibrio vulnificus by Alternative Carbon Sources
Source: Int J Mol Sci. 2022 Dec 3;23(23):15278. doi: 10.3390/ijms232315278 (PMC9737408; doi:10.3390/ijms232315278)
Supplement: Supplementary file 1 [file ijms-23-15278-s001.zip › ijms-2057164-supplementary.pdf]

Table S1: RT-qPCR Primers. Gene name, primer pairs and corresponding GeneBank accession number of the selected genes used. All the primers were designed in this study.

Gene name	Primer pairs sequence	Accession number
hlyU	GGTCGGGGAACTTTGTGC<br>CCGTTTGTGCTTCTTTACGC	DI002108.1
vvhA	CGTGACCTACCGCAGAATG<br>GACTTCGCCACCCACTTTC	KC821520.1
vvpE	GCTCTGCGATGACATTTGG<br>GCCTCCCGACATATTGCTG	FJ864304.1
rtxA	ATCCAGAAACGGGCAAACTC<br>TCAAAACCACCAGCAGAAGAG	FJ002581.2
plpA	CTGATGACCTTGCCTGATGCTAC<br>CGCTTGTGCTTTGATGAACTC	CP002470.1
aphB	CACCCGACGCATACAAGC<br>TGACGCACCACGACATTC	CP014636.1
smcR	TGGCGACCGTCTTCAACTAC<br>TGGCGATGTTTTCTTTAGCG	AF204737.1
lrp	CGTAAATTGCTCGGTGATACTCTG<br>GGTTGGTTTGCTTCACTTCTTCC	AY160773.1
hupA	TTAACTCCTGGCGTTCGTTAC<br>TCTTGCGTTACCTTGTAAGTGG	KC741523.1
VVA1308	CTGGTTTTTGGCTGTAGATGG<br>CTTAGACGCTCTGGATTATCTTGG	CP044068.1
VvdRP	AGGATGAGTCCGTTTATCGCC<br>GCAGATGCTAAACCTTTCATAACTATCAC	CP012881.1
vvuA	TGTTTGGTGCTCGCTCTAGTTC<br>CTAACGGTGATGTGCTTATCGC	BA000038.2
gyrB	GATGCGCGTGAAGGTTTG<br>CTGCCATGAAGGATTCCAC	GQ382205.1
recA	GCCGCATTGACACCAAAG<br>TCACGCCGATCTTCATACG	GQ382239.1
